# Molecular modifications to mitigate oxidative stress and improve red blood cell storability

**DOI:** 10.3389/fphys.2024.1499308

**Published:** 2024-10-30

**Authors:** Alkmini T. Anastasiadi, Konstantinos Stamoulis, Anastasios G. Kriebardis, Vassilis L. Tzounakas

**Affiliations:** ^1^ Department of Biochemistry, School of Medicine, University of Patras, Patras, Greece; ^2^ Hellenic National Blood Transfusion Centre, Acharnes, Greece; ^3^ Laboratory of Reliability and Quality Control in Laboratory Hematology (HemQcR), Department of Biomedical Sciences, School of Health and Welfare Sciences, University of West Attica (UniWA), Egaleo, Greece

**Keywords:** red blood cells, storage lesion, oxidative stress, blood transfusion, storage improvement, antioxidant enhancement, hypoxia, proteasome

## Abstract

The development of red blood cell (RBC) storage lesion during hypothermic storage has long posed challenges for blood transfusion efficacy. These alterations are primarily driven by oxidative stress, concern both structural and biochemical aspects of RBCs, and affect their interactions with the recipient’s tissues post-transfusion. Efforts to counteract these effects focus on improving the antioxidant capacity within stored RBCs, reducing oxygen exposure, and scavenging harmful molecules that accumulate during storage. Various supplements, such as ascorbic acid, N-acetylcysteine, polyphenolic compounds, and specific metabolites have shown the potential to improve RBC quality by reducing oxidative lesions and lysis phenomena, and enhancing antioxidant, energy, or proteostasis networks. Accordingly, anaerobic storage has emerged as a promising strategy, demonstrating improved RBC storability and recovery in both animal models and preliminary human studies. Finally, targeted scavenging of harmful storage-related phenotypes and molecules, like removal signals, oxidized proteins, and extracellular hemoglobin, while not so studied, also has the potential to benefit both the unit and the patient in need. Omics technologies have aided a lot in these endeavors by revealing biomarkers of superior storability and, thus, potential novel supplementation strategies. Nonetheless, while the so far examined storage modifications show significant promise, there are not many post-transfusion studies (either *in vitro*, in animal models, or humans) to evaluate RBC efficacy in the transfusion setting. Looking ahead, the future of blood storage and transfusion will likely depend on the optimization of these interventions to extend the shelf-life and quality of stored RBCs, as well as their therapeutic outcome.

## 1 Oxidation-driven storage lesion and its post-transfusion impact

### 1.1 Oxidative stress as a driver of storage lesions in red blood cells

During their hypothermic storage, red blood cells (RBCs) accumulate a wide array of defects, collectively known as storage lesion. The primary culprit is oxidative stress, where hemoglobin (Hb) oxidation (Yoshida et al., 2019) leads to the formation of methemoglobin (metHb) and superoxide anions (O_2_^•−^). Low temperatures accelerate metHb denaturation, releasing free hemin or heme and forming hemichromes (Samuel et al., 2020), which tightly bind to the membrane, increasing rigidity, promoting removal signals, like band three aggregates (Badior and Casey, 2018), and disrupting phosphorylation events (Pantaleo et al., 2016). At the same time, Fenton and Haber-Weiss reactions can produce hydroxyl radicals (HO^•^), one of the most potent oxidizing radicals in biology (Moller et al., 2023). While superoxide anions are not toxic *per se*, they can react with nitric oxide (NO^•^) to produce peroxynitrite (ONOO^−^) (Cortese-Krott, 2023), which further reacts with CO_2_ to generate powerful oxidants like nitrogen dioxide (NO_2_^•^) and carbonate radical (CO_3_^•−^) (Denicola et al., 1996). All these radicals attack cellular targets, leading to lipid peroxidation and protein carbonylation, initiating a cascade of cellular and biochemical alterations. Hb is one of the main targets of oxidative stress, further propagating the production of radical species. Additionally, ferrylHb is formed and exhibits extremely high redox potential, rendering it highly toxic to cellular molecules (Alayash, 2022). In terms of RBC integrity, structural proteins also undergo oxidation (D'Alessandro et al., 2012), and many oxidized molecules migrate to the membrane, disrupting its connections with the cytoskeleton and reducing RBC deformability (Barshtein et al., 2021). These changes affect phospholipid distribution in the membrane, exposing phosphatidylserine (PS), a marker of cell removal (Koshkaryev et al., 2020). In parallel, the “do-not-eat-me” signal of CD47 is also converted to an “eat-me” signal through oxidatively induced conformational changes (Burger et al., 2012). Moreover, several bioactive products of oxidation or glycoxidation processes, including oxylipins and advanced glycation end products (AGEs), accumulate during storage (Lysenko et al., 2006; Fu et al., 2016). The oxidation of metabolic enzymes, combined with acidic conditions, reduces key metabolites like 2,3-diphosphoglycerate (2,3-DPG), ATP, and NADPH. At the same time, systems that redirect glucose to the pentose phosphate pathway to enhance reducing power in stored RBCs (Rinalducci et al., 2015) become less effective due to fragmentation of the N-terminus of band 3, which regulates metabolic fluxes (Rinalducci et al., 2012; D'Alessandro et al., 2023). Finally, ATP depletion and low temperatures restrict Na^+^-K^+^ ATPase functionality, altering the cation gradient across the membrane (Flatt et al., 2014), and affecting RBC volume and shape (Zimna et al., 2021).

**FIGURE 1:**
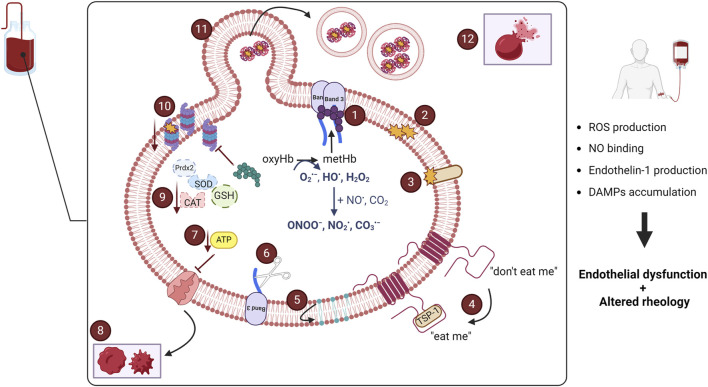
Hallmarks of stored red blood cell oxidative lesions. Oxyhemoglobin (OxyHb) oxidation leads to the generation of methemoglobin (metHb) and reactive oxygen species (ROS). MetHb can form hemichromes that get attached to band 3 (1) and aggravate red blood cell (RBC) deformability. ROS can fuel the production of reactive nitrogen species and altogether oxidize lipid (2) and protein (3) molecules. All these phenomena aid in the emergence of removal signals, by modifying self-antigens, like CD47 (4), or exposing phosphatidylserine on the RBC surface (5). The fragmentation of the cytosolic N-terminus of band 3 (6) alters metabolic fluxes, while ATP depletion (7) affects the normal function of ion channels, and thus, ion homeostasis, ultimately leading to shape modifications (8). At the same time, both enzymatic and non-enzymatic antioxidant mechanisms are compromised (9), the proteasome machinery’s activity is reduced (10), and the cell sacrifices part of its membrane through vesiculation to remove oxidized molecules (11). Altogether, these lesions lead to the occurrence of hemolysis (12), the gold standard of blood storage. CAT: catalase; DAMP: damage-associated molecular pattern; GSH: glutathione; NO: nitric oxide; prdx2: peroxiredoxin 2; SOD: superoxide dismutase; TSP-1: thrombospondin 1. Created in BioRender.com/f93h148.

Despite being equipped with an antioxidant defense system, including enzymes like superoxide dismutase, peroxiredoxin 2, and catalase, and non-enzymatic scavengers like glutathione (GSH) and ascorbate (Moller et al., 2023; Chatzinikolaou et al., 2024), stored RBCs are characterized by redox imbalance. Many antioxidant enzymes become oxidized (Delobel et al., 2016), as in the case of peroxiredoxin 2; its oxidized form is accumulated during storage due to its decreased reduction (Sadowska-Bartosz and Bartosz, 2023). Accordingly, GSH gets depleted, especially since its *de novo* synthesis is minimal (Whillier et al., 2011; D'Alessandro et al., 2017). Additionally, the oxidation of membrane-related molecules and translocation of oxidized proteins to the membrane creates a source of reactive species that is less accessible to the cytosolic antioxidant system (Mohanty et al., 2014). Proteasomal degradation of carbonylated proteins can help alleviate cellular stress (Delobel et al., 2012), but proteasomal activity decreases in the cytosol during storage (Anastasiadi et al., 2021; Peltier et al., 2024). Although proteasome’s subunits translocate to the membrane around the middle of the storage period, their activity declines later, likely due to accumulated defects (Tzounakas et al., 2022c) or inability to process overoxidized aggregates (Delobel et al., 2012). Another way of dealing with damaged molecules is their compartmentalization and release through extracellular vesicles (EVs). While this ostracization of potentially toxic molecules is helpful, it exacerbates membrane integrity issues, altering RBC morphology and leading to the ultimate storage lesion phenotype: hemolysis (Figure 1) (Melzak et al., 2021; Tzounakas et al., 2022b).

### 1.2 Interaction of stored RBCs with recipient tissues post-transfusion

The extensive antioxidant system of RBCs can benefit every plasma-accessible part of the organism since redox buffering is one of the many altruistic functions of these cells (Anastasiadi et al., 2024a). This is possible through transmembrane electron transport, which allows the reduction of extracellular oxidants. Potential cytosolic sources of reducing equivalents include NADH, ascorbate, and flavonoids, while the plasma membrane redox system of RBCs seems to be comprised of distinct oxidoreductases, with the involvement of cytochromes and sulfhydryl groups (Matteucci and Giampietro, 2007). A notable example concerns the recycling of ascorbic acid. RBCs can retrieve dehydroascorbate from their environment and convert it to ascorbic acid. This boosts the plasma membrane electron transport through the duodenal isoform of cytochrome b561, the main substrate of which is the extracellular ascorbate free radical, reducing it back to ascorbic acid (Eigenschink et al., 2021). The disturbance of RBC redox potential during storage, along with the elevated lysis and subsequent release of prooxidant molecules in the circulation post-transfusion can have detrimental consequences on systemic redox signaling, especially in the vasculature. Reactive oxygen species (ROS) regulate vascular function, but they must be within specific limits to remain harmless (Obradovic et al., 2020). Redox disturbance in the endothelium can also be caused by AGEs that accumulate on the surface of stored RBCs, since they can interact with their receptor in the microvasculature, induce ROS production, and deplete antioxidant powers (Mangalmurti et al., 2010).

It should not be omitted that the interaction of RBCs with vascular function is predominantly through nitric oxide (NO) metabolism. NO is a powerful vasodilator that initiates a cascade that relaxes smooth muscle by lowering intracellular calcium (Zhao et al., 2015). RBC membrane is believed to possess functional domains, one of which is related to NO production via the interaction of deoxyHb with nitrite (Gladwin et al., 2005). Additionally, RBCs are equipped with an active endothelial NO synthase that converts L-arginine to citrulline, releasing NO in the process (Cortese-Krott and Kelm, 2014), a reaction that is affected by elevated oxidative stress (Cortese-Krott and Shiva, 2019). Yet, both free and vesicular Hb can capture NO in the circulation upon transfusion, limiting its bioavailability (Buehler and Alayash, 2004) and potentially decreasing blood flow in the microcirculation, ultimately leading to organ compromise (Roback, 2011; Weinberg and Patel, 2016). In the same context, AGEs have been also shown to quench NO and at the same time induce endothelin-1 (a powerful vasoconstrictor), further tipping the scales in favor of vasoconstriction (Basta et al., 2004). In addition, *in vitro* experiments support that PS-exposing RBCs can be targeted by secretory phospholipase A_2_, releasing lysophosphatidic acid, a potent lipid mediator that can induce endothelial dysfunction and even vascular leak (Neidlinger et al., 2006). Finally, oxylipins are also linked to hemodynamic properties post-transfusion, probably explaining the increase in blood flow even when NO synthesis is compromised (Figure 1) (D'Alessandro et al., 2019).

## 2 Counteracting oxidative stress in the blood bank

**FIGURE 2:**
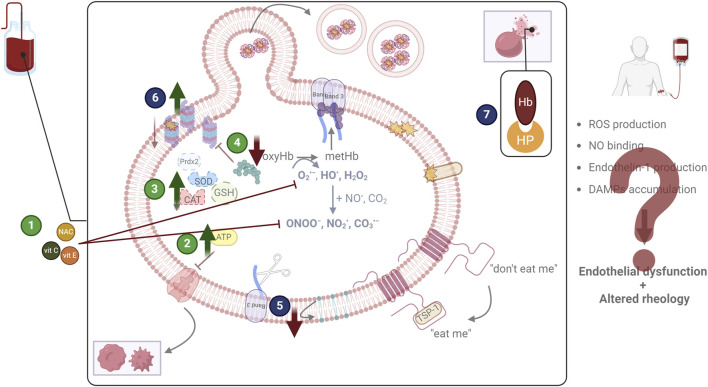
Examined and proposed interventions to address oxidative lesions. The most widely tested storage modification regards the addition of antioxidant molecules (1) to scavenge reactive species and inhibit their detrimental effects. Other approaches focus on the enhancement of metabolism (either energy or redox) (2), or of antioxidant enzymes (3), again to counteract oxidative stress. Finally, the reduction of oxygen (4) by storing red blood cells in anaerobic conditions is also being examined since it targets the root of the problem. Apart from addressing oxidative stress (green circles), other efforts aim at diminishing the lesions that emerge as its consequence (blue circles). The decrease of removal signals (5), the enhancement of proteostasis (6), and the scavenging of damage-associated molecular patterns (DAMPs), including hemoglobin (Hb) through haptoglobin (HP) addition (7) have been discussed. CAT: catalase; GSH: glutathione; NAC: N-acetyl cysteine; NO: nitric oxide; prdx2: peroxiredoxin 2; ROS: reactive oxygen species; SOD: superoxide dismutase; TSP-1: thrombospondin 1; vit: vitamin. Created in BioRender.com/q76t533.

Upon recognizing the relation between oxidative stress and storage lesions and by utilizing elegant omics technologies (Nemkov et al., 2024b; Thomas et al., 2024) and advanced platforms for the evaluation of multiple storage strategies (Nemkov et al., 2022), blood transfusion research has increasingly focused on discovering biomarkers of superior storability and refining storage conditions to mitigate oxidative events. Addressing such lesions holds the promise of not only extending the quality and shelf-life of blood units, but also of reducing adverse post-transfusion reactions to improve patient outcomes, paving the way for precision blood transfusion, where blood units and distinct storage strategies can be selected to match specific patient cohorts. In this context, three main routes have been explored: the enhancement of extracellular and intracellular antioxidant defenses, the reduction of oxygen, and the targeted scavenging of harmful biological molecules (Figure 2).

### 2.1 Forming the basis: antioxidant and metabolic enhancement

The most studied antioxidant supplement in stored RBCs is ascorbic acid, with human studies indicating that it attenuates oxidative stress and lesions (Bardyn et al., 2021; Tzounakas et al., 2022a) and boosts antioxidant defenses (Anastasiadi et al., 2023), as also supported by the elevated protein sulfhydryls in stored RBCs from rats (Ravikumar and Rajashekaraiah, 2017). However, results regarding hemolysis are contradictory (Raval et al., 2013; Czubak et al., 2017). Notably, supplemented murine RBCs present better recovery (Stowell et al., 2013), something not evident upon transfusion in xenobiotic models (Tzounakas et al., 2022a). When in combination with uric acid there is an improvement in terms of redox equilibrium, metabolic lesions (Bardyn et al., 2020), and proteostasis (Tzounakas et al., 2022a). Another molecule that has been examined in combination with ascorbic acid is N-acetylcysteine (NAC), a precursor of GSH, leading to reduced hemolysis and ROS accumulation, and improved antioxidant metabolism (Pallotta et al., 2014). Similar results also emerge upon the sole addition of NAC (Amen et al., 2017). Combining vitamins C and E in stored rat RBCs does not seem to alter their storability features (Pallavi and Rajashekaraiah, 2023); yet, vitamin E alone efficiently reduces ROS and hemolysis in stored RBCs from healthy humans (Silva et al., 2017; Nemkov et al., 2022). While most studies focus on the addition of antioxidants in the stored units, the antioxidant “treatment” of donors some days before blood donation, also has the potential to provide RBCs with improved storability (Addis and St Cyr, 2017; Kim et al., 2022). Such is the case of taurine, which boosts the antioxidant metabolism of stored RBCs both after supplementation of the donors and of the units, and results in higher post-transfusion survival rates in mouse models (Bertolone et al., 2020).

Other potentially beneficial supplements are derived from plant extracts. For instance, resveratrol can induce the activity of antioxidant enzymes and the levels of GSH in stored RBCs, protecting them from oxidative insults (Huyut et al., 2018). The fact that the same compound upregulates the plasma membrane redox system of RBCs (Pandey and Rizvi, 2013) could also benefit the patients post-transfusion. Similar improvements are seen when caffeic acid (Huyut et al., 2016) or astaxanthin are added in packed RBCs, but in the second case, there is an additional reduction in hemolysis and ROS accumulation (Wang et al., 2015; Wang and Wang, 2021), as also seen when naringin is used (She et al., 2022). Recently, the supplementation of stored RBCs with curcumin improved ATP preservation and slowed oxidative lesion emergence. However, the most promising outcomes concern post-transfusion aspects: a drop was observed in fibrinogen subunit levels that could lead to minor aggregation, while recovery was increased, satisfying the gold standard of transfusion therapy (Hicks et al., 2024). Antioxidant supplementation can also be used in distinct settings. In the context of γ-irradiated RBCs, the presence of quercetin seems to partly improve some redox parameters, but at the same time exacerbates hemolysis phenomena (Zbikowska et al., 2014). Such interventions could be useful for targeted supplementation of units from donor cohorts with compromised redox metabolism (Tzounakas et al., 2016; Hazegh et al., 2021).

Metabolites could not be absent from such a list. Since GSH rapidly decreases during storage, its direct addition has a protective effect (Dumaswala et al., 2000), whereas the addition of its precursors only slightly elevates its levels (Whillier et al., 2011; D'Alessandro et al., 2017). Based on the ability of RBCs to synthesize NAD^+^ from nicotinic acid, supplementing them with the latter protects them from oxidation and lysis by increasing intracellular antioxidants (Arun et al., 1999). Similar interventions have been made to maintain adequate levels of 2,3-DPG and ATP, as the ones using phosphate or pyruvate (Oski et al., 1971; Dawson et al., 1981). In the same context, since sphingosine-1-phosphate promotes glycolysis, units supplemented with it better maintain their energy, but at the cost of reducing agents, rendering them inferior regarding post-transfusion recovery (Hay et al., 2023b). Supporting energy metabolism also affects redox dynamics, as shown by the improvement of antioxidant defenses and the minor post-transfusion liver oxidation and inflammation after supplementation of stored murine RBCs with sodium pyruvate (Xia et al., 2016). Moving on to lipid metabolism, L-carnitine is one of the molecules that stand out due to its role in repairing oxidized membrane lipids. Indeed, its addition to stored RBCs protects them from oxidative lesions (Ravikumar et al., 2020), enhances their resilience to lysis (Arduini et al., 1997), and leads to superior recovery and Hb increment post-transfusion (Nemkov et al., 2024a). Again, the consumption of such metabolites by the donor could also benefit their stored RBCs. To support this, stored RBCs from mice fed with diets enriched in long-chain polyunsaturated fatty acids −molecules that can support RBC membrane properties− are characterized by improved deformability and lipid peroxidation, and a boosted post-transfusion survival (Kim et al., 2022; Kim et al., 2023).

Finally, the addition of antioxidant enzymes, albeit understudied, is also interesting. Barzegar et al., developed nanoparticles containing two of the main RBC antioxidant enzymes, superoxide dismutase, and catalase, to counteract the drop in activity that is observed during storage. Their presence in the blood bag attenuates the observed increase in oxidative stress and morphology deterioration (Barzegar et al., 2021; Barzegar et al., 2022). The use of small molecules that mimic the activity of enzymes has been also examined *in vitro*. More specifically, selenium-based peroxiredoxin mimetics have been suggested to protect RBCs from oxidative insults and the emergence of eryptotic phenotypes (Chakrabarty et al., 2020). Another promising approach concerns the modulation of the expression of antioxidant enzymes in RBC precursors. Recently, reticulocytes with enhanced peroxiredoxin and glutathione peroxidase proteins were successfully engineered, giving hope for the potential to improve RBC storability (Langlands et al., 2024).

### 2.2 Adding new perspectives:oxygen reduction

Anaerobic storage has emerged as an alternative to normoxic conditions that could potentially enhance RBC quality by removing oxygen at the beginning of storage and maintaining the hypoxic state throughout its duration. The outcomes so far are promising, showing improved storability and beneficial post-transfusion outcomes. For instance, under hypoxic storage RBCs are rendered metabolically superior, since they better maintain their 2,3-DPG levels, are characterized by improved GSH homeostasis, and present minor purine oxidation (Meng et al., 2019; D'Alessandro et al., 2020). This metabolic rewiring is also linked to better oxygen-unloading kinetics (Rabcuka et al., 2022). Oxidative stress is indeed mitigated since anaerobic RBCs accumulate less ROS during their storage (Bencheikh et al., 2022), and at the same time, they better retain their morphology and integrity (Meng et al., 2019). All these findings corroborate the potential of storage extension by using this strategy (Yoshida et al., 2007). Investigations in mice report similar storability findings and take it a step further by stating the superior post-transfusion survival (Hay et al., 2023a) and efficacy (Williams et al., 2020) of hypoxic RBCs. Notably, *in vitro* models of transfusion hint at the possibility of better performance in sickle cell transfusion recipients (Karafin et al., 2023), while autologous transfusion events are linked to improved RBC recovery when hypoxic storage is used (D'Alessandro et al., 2020). Recently an interim report, regarding the outcomes of anaerobically stored RBC administration to patients with hematologic malignancies, demonstrated the tolerance of these cells by the patients and gave hope for increasing the window between consecutive transfusions (Reikvam et al., 2023).

### 2.3 Introducing a different focus: molecular targeting

Instead of targeting oxidative stress, one could focus on its outcomes. Such an example is the addition of erythropoietin (Epo) in RBC units. Epo interacts with erythroid precursors and promotes their viability and differentiation. There are indications for mature RBCs to retain some Epo receptors, and therefore Epo supplementation could better retain RBC viability in the unit; indeed, the emergence of removal signals was mitigated (Penuela et al., 2016). Similar was the aim of the study of Hoehn et al., in which acid sphingomyelinase, an enzyme that aids in the emergence of removal signals on RBCs, was inhibited during storage (Hoehn et al., 2016). With this approach, the integrity of RBCs was improved, and less PS was exposed on their surface, while at the same time, the release of Hb in the circulation of the recipient was minor. Another potential molecular target is the proteasome machinery. Based on its role in decongesting stored RBCs from accumulating oxidized proteins, and the decline in its activity during storage, its enhancement has been suggested as a promising approach to counteract storage lesion (Delobel et al., 2016; Anastasiadi et al., 2023). Preliminary observations of our team, indeed support that upregulated proteasome can benefit stored RBCs, since donor cohorts of high proteasomal activity are characterized by an improved RBC storability profile in terms of lysis and oxidation parameters (Anastasiadi et al., 2024b). Other approaches focus on the extracellular compartment to reduce the accumulating damage-associated molecular patterns (DAMPs), since they are harmful both to stored RBCs, as well as to the recipient. A delicate attempt was recently made with the use of nanofibrous sheets that were able to scavenge DAMPs and slow the integrity lesions of stored RBCs. Importantly, this intervention also positively affected the post-transfusion survival of RBCs in mice models (Pandey et al., 2022). Since free Hb is one of the most abundant and potent DAMPs, the addition of haptoglobin in the units has been discussed (Wang et al., 2014). If effective, such supplementation could protect the patient from unwanted free Hb-related outcomes, as in the case of NO binding and ROS production, but its applicability and success remain to be determined.

## 3 Considerations and future perspectives

Oxidative stress is a pivotal factor in the development of RBC storage lesions, leading to a cascade of cellular and biochemical alterations that compromise quality and function. Addressing these lesions by enhancing antioxidant defenses, reducing oxygen levels, and scavenging harmful molecules shows promise in improving the storability and efficacy of stored blood. Omics technologies and biomarker identification offer a pathway to optimizing storage conditions, ultimately leading to better patient outcomes. However, while the potential for these strategies is promising, the field still lacks comprehensive studies on post-transfusion efficacy and adverse effects. Current data are limited to *in vitro* models and animal studies, with few clinical trials providing conclusive results. To bridge this gap, more studies should focus on pre-clinical evaluations, moving towards clinical trials, and eventually integrating these findings into clinical practice. Like a story yet to be written, the future of blood storage and transfusion will require a deeper understanding of the complex interplay of oxidative stress, storability, and transfusion outcomes.
